# Efficacy of Aspirin in Preventing Venous Thromboembolism in Total Knee and Hip Joint Replacement

**DOI:** 10.7759/cureus.74347

**Published:** 2024-11-24

**Authors:** David Campos-Flores, Andrés Mercado-Arce, Alí Gómez-González, Julio Flores-Rascón, Fernando Williams-Manzo, Roberto Ramos-Tercero, Jose Luis Gálvez-Romero, Ana Luisa Galicia-Zamalloa

**Affiliations:** 1 Orthopaedics and Traumatology, Instituto de Seguridad y Servicios Sociales para los Trabajadores del Estado, Puebla, MEX; 2 Orthopedics and Traumatology, Instituto de Seguridad y Servicios Sociales para los Trabajadores del Estado, Puebla, MEX; 3 Biostatistics, Instituto de Seguridad y Servicios Sociales para los Trabajadores del Estado, Puebla, MEX

**Keywords:** aspirin therapy, thromboprophylaxis, total hip replacement (thr), total knee replacement (tkr), venous thromboembolsim

## Abstract

Background: Venous thromboembolism (VTE) is a significant complication following total knee arthroplasty (TKA) and total hip arthroplasty (THA). Aspirin has gained attention as a cost-effective, safe alternative to traditional anticoagulants like enoxaparin, but comparative data on efficacy and safety remain limited.

Methods: This randomized controlled trial compared the efficacy of aspirin and enoxaparin in preventing VTE following TKA and THA. A total of 60 patients were recruited, with 30 patients in each group. Prophylactic efficacy was assessed through Doppler ultrasonography preoperatively and at two and four weeks postoperatively. The primary outcome was the incidence of VTE or bleeding events within 90 days.

Results: No cases of symptomatic deep vein thrombosis, pulmonary embolism, or other thrombotic events were observed in either group during the 90-day follow-up. Additionally, no statistically significant differences were noted between groups for bleeding events, including hematoma or hemarthrosis. Both treatments were well tolerated, with comparable safety profiles. Systemic and gastrointestinal complications were minimal and evenly distributed across groups.

Conclusion: Our findings suggest that aspirin and enoxaparin are equally effective in preventing VTE after total knee or hip arthroplasty. Further research is needed to determine the optimal duration and dosage of aspirin for VTE prophylaxis in this setting.

## Introduction

Total hip arthroplasty (THA) and total knee arthroplasty (TKA) are widely performed and effective procedures for patients with degenerative joint diseases, such as osteoarthritis, aiming to restore function and alleviate pain. However, venous thromboembolism (VTE), encompassing deep vein thrombosis (DVT) and pulmonary embolism (PE), poses a significant postoperative risk. This complication is associated with considerable morbidity and, in some cases, mortality, alongside substantial healthcare costs for long-term management surgical duration and limited perioperative mobility in TKA and THA patients, further elevating VTE risk, leading to routine anticoagulant use for up to 35 days postoperatively in most cases [1-5].

Althoporary protocols and multimodal approaches have substantially reduced VTE incidence, determining the most effective chemoprophylactic agent remains a point of debate. While low-molecular-weight heparin (LMWH) and newer oral anticoagulants, such as rivaroxaban and dabigatran, offer effective prophylaxis, these agents also carry higher costs and potentially greater bleeding risks, including major hemorrhagic events and wound complications [5-8].

Aspirin, a cost-effective agent, has gained popularity for VTE prevention in arthroplasty patients. Its safety profile, ease of administration, and absence of monitoring requirements make it a favorable choice. Retrospective studies and observational data from 2010 to 2021 support its efficacy in reducing VTE incidence in joint replacement [9-13].

Furthermore, low-dose aspirin is comparable to higher doses in preventing cardiovascular and cerebrovascular events and may even be more effective in certain cases [13,14]. Given the dose-dependent toxicity of aspirin, particularly the risk of gastrointestinal bleeding [15], determining the optimal dose for VTE prevention remains an area of investigation.

To date, limited evidence exists to support the use of low-dose aspirin for treating VTE after TKA. This study aimed to evaluate the efficacy of aspirin in preventing venous thromboembolism during total knee and hip joint replacement.

## Materials and methods

Ethical approval

The trial was approved (Approval number: 151.2022) by the research ethics committee of the Instituto de Seguridad y Servicios Sociales de los Trabajadores del Estado (ISSSTE) Hospital Regional Puebla in Mexico. It was conducted following the principles of the Declaration of Helsinki. All patients provided written informed consent before the start of the study. 

Trial registration: ClinicalTrials.gov, NCT06635317. Registered 10 October 2024, https://clinicaltrials.gov/study/NCT06635317

Study population

All patients were enrolled between April and October 2022 in the Instituto de Seguridad y Servicios Sociales de los Trabajadores del Estado (ISSSTE) Hospital Regional Puebla in Mexico and were allocated into two groups following a 1:1 ratio. This enrollment process was conducted according to the inclusion and exclusion criteria described below. Computer randomization was used to develop the allocation sequence by a data manager who was not responsible for assigning participants to their respective treatment groups. After surgery, a different data manager assigned the corresponding prescription.

For the sample size calculation, we employed a non-inferiority clinical trial design. Based on prior research analysis comparing symptomatic VTE incidence is 4% after THA or TKA [16], a statistical power of 80% power with a non-inferiority margin of 1%. The calculated sample size required a minimum of 30 patients in each treatment group to achieve adequate statistical power.

Eligibility criteria

We included patients with primary total hip or total knee arthroplasty for osteoarthritis. We excluded patients considered to have high risk for VTE (IMPROVE Bleeding score greater than or equal to 7, direct oral anticoagulant, dual antiplatelet therapy, and medical contraindications (allergy, cancer, cardiac disease, kidney disease, or bleeding disorder precluding anticoagulation). 

Study design

All patients included in this study underwent a preoperative Doppler ultrasound of the lower extremities to screen for prothrombotic risk. One experienced orthopedic surgeon performed the THA using the posterior-lateral approach and TKA using the install approach, both under spinal anesthesia. Surgical techniques and prosthetic selection were determined at the discretion of the attending surgeon. Antibiotic prophylaxis consisted of a 1-gram dose of cephalotin administered intravenously. No surgical drains were used in this study.

After surgery, patients received 5 weeks of thromboprophylaxis with either aspirin or enoxaparin control. At six hours post-surgery, patients in the aspirin group received enoxaparin at a dose of 40 mg daily, administered subcutaneously for three days, followed by oral aspirin at a dose of 100 mg daily to complete the 5 weeks. Whereas patients in the enoxaparin control group received 40 mg of enoxaparin daily via subcutaneous injection to complete the 5 weeks; for those weighing less than 50 kg or with an estimated glomerular filtration rate (eGFR) below 30 mL/min/1.73 m², the enoxaparin dose was reduced to 20 mg daily to mitigate bleeding risk. Postoperative care included the use of pneumatic compression stockings for a minimum of 21 days as standard thromboprophylaxis.

Additionally, physical therapy was initiated on the day of surgery or by postoperative day one, with daily sessions continuing throughout the hospital stay to enhance functional recovery. Postoperative wound care and Doppler ultrasound assessments were conducted at two and four weeks after surgery to monitor for any signs of VTE.

Data collection and outcomes

Primary Outcomes

Patient demographic characteristics, including age, gender, body mass index, and medical history, were all recorded on admission.

The primary outcome of this study was the 90-day incidence of postoperative bleeding events and prothrombotic complications. Bleeding events encompassed hematoma, hemorrhage, acute postoperative blood loss anemia, hemarthrosis, and the rate of allogeneic blood transfusion. Prothrombotic complications included the occurrence of DVT, PE, stroke, and myocardial infarction (MI). Patients were instructed on identifying and reporting potential VTE symptoms. DVT diagnosis and confirmation were conducted through Doppler ultrasonography, performed preoperatively and during follow-up between postoperative days 14 and 30, specifically examining the deep ascending veins (popliteal, femoral, common femoral, and iliac) for emboli. Additionally, measurements of thigh and calf circumferences were taken pre-and postoperatively.

All postoperative complications were documented and classified into categories: surgery-related complications (such as surgical site infection, periprosthetic joint infection, and hip dislocation), systemic adverse events (including myocardial infarction, stroke, and death), and gastrointestinal (GI) side effects.

Statistical analysis

Patient demographic characteristics, comorbidities, type of joint replacement, and implant type were recorded via Microsoft Excel. All statistical tests were performed using SPSS Statistics (software version 25, IBM Corporation, Armonk, USA). The rate difference and 95% confidence interval (CI) were utilized in the non-inferiority analysis of postoperative VTE incidence. Quantitative data were presented as medians and interquartile ranges, with inter-group and intra-group comparisons conducted using the Mann-Whitney U-test and Friedman test, respectively. For qualitative data comparisons, the chi-square test was applied. Univariable and multivariable logistic regression analyses were performed to identify and control for confounding factors associated with DVT incidence. A p-value of less than 0.05 was considered statistically significant across all tests.

## Results

**Figure 1:**
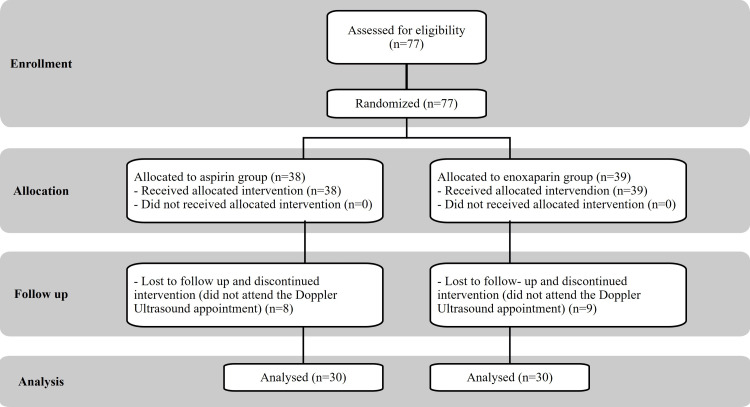
Flowchart showing the randomized distribution of enrolled patients into groups prescribed with aspirin or enoxaparin.

In this study, we analyzed 60 patients (48 (80%) who underwent TKA and 12 (20%) who underwent THA) between April and October 2022. After the screening for eligibility, 77 patients were randomized; 38 were allocated to the aspirin group and 39 to the enoxaparin group. Seventeen patients were lost to follow-up from the study before its completion (Figure 1). Baseline demographic data for patients in the VTE prophylaxis subgroup are listed in Table 1. The mean age of the patients was 68.52 years (44-85 years); 43 (71.1%) of the patients were women, and 17 (28.3%) were men. Among the most frequently reported comorbidities in this study, hypertension emerged as the primary condition, affecting 55% of patients. Obesity was the second most prevalent comorbidity at 41.7%, followed by type 2 diabetes mellitus at 30% and rheumatoid arthritis at 11.7% of patients.

**Table 1:** Baseline demographic data TKA: Total knee arthroplasty, THA: Total hip arthroplasty, ST: Standard deviation, BMI: Body mass index.

	n= 60 (100)
	N (%)
Groups	
Aspirin group	30 (50)
Enoxaparin group	30 (50)
Gender	
Female	43 (71.7)
Male	17 (28.3)
Comorbidities	
Hypertension	33 (55)
Diabetes	18 (30)
Rheumatoid arthritis	7 (11.7)
Obesity	25 (41.7)
Surgery	
TKA	48 (80)
THA	12 (20)
Age (x ± SD)	68.5 ± 8.6
BMI kg/m^2^ (x ± SD)	28.8 ± 4.5

At the time of analysis (Table 2), 30 patients received aspirin prophylaxis, of whom 8 were male and 22 were female. In the enoxaparin group, nine patients were male, and 21 were female. Among the procedures performed, 25 TKAs and five THAs were recorded in the aspirin group, whereas the enoxaparin group included 23 TKAs and seven THAs. Regarding obesity, 13 patients in the aspirin group and 12 in the enoxaparin group had obesity, totaling 25 patients. In terms of comorbidities, eight patients in the aspirin group and 10 in the enoxaparin group had a history of type 2 diabetes, resulting in a total of 18 patients with this condition.

**Table 2:** Analysed groups OR: Odds ratio; IC: Information coefficient; TKA: Total knee arthroplasty; THA: Total hip arthroplasty

	Aspirin group n=30 (%)	Enoxaparin group n=30 (%)	OR (IC_95%_)	* p
Gender				
Female	22 (73.3)	21 (70)	0.8 (0.3-2.6)	0.7
Male	8 (26.6)	9 (30)		
Surgery				
TKA	25(83.3)	23 (76.6)	1.5 (0.4-5.4)	0.5
THA	5 (16.6)	7 (23.3)		
Comorbidities				
Obesity	13 (43.3)	12(40)	0.9 (0.3-2.4)	0.8
Diabetes	8 (26.6)	10 (33.3)	1.3 (0.5-4.1)	0.5
Hypertension	15 (30)	18 (60)	1.5 (0.5-4.1)	0.4
Rheumatoid arthritis	4 (13.3)	3 (10)	0.7 (0.1-3.5)	0.6

Additionally, 15 patients in the aspirin group and 18 in the enoxaparin group had a history of hypertension, giving a total of 33 patients with this comorbidity. For rheumatoid arthritis, four patients in the aspirin group and three in the enoxaparin group had a history of this condition, totaling seven patients. Importantly, there were no reported surgical site complications in either study group through the outpatient follow-up period, including until suture removal and wound healing were complete.

**Figure 2:**
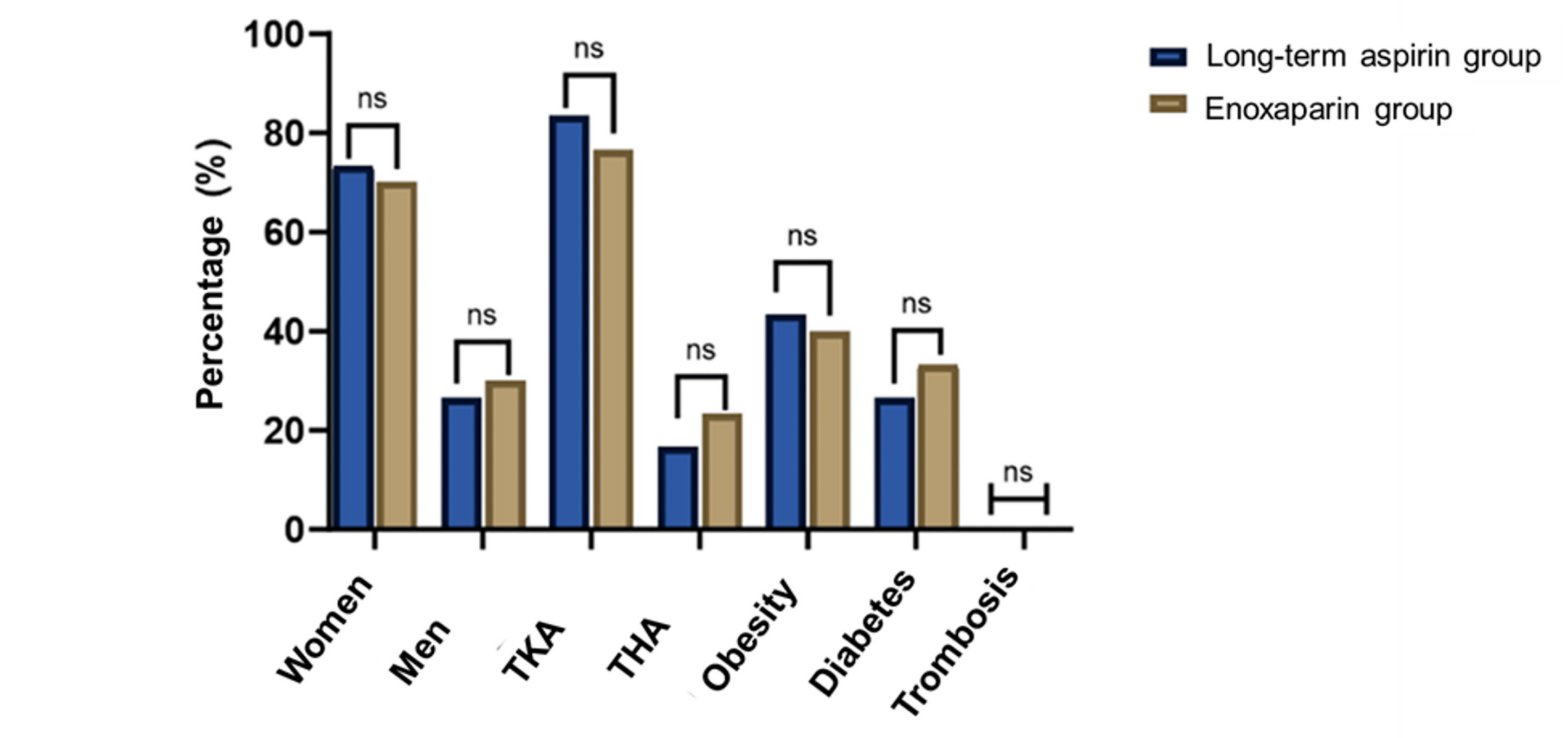
A comparative analysis of the efficacy of aspirin and enoxaparin in preventing thrombosis in patients undergoing knee or hip replacement was performed TKA: Total knee arthroplasty, THA: Total hip arthroplasty, NS: Non-significant

In both groups, no patients were diagnosed with symptomatic VTE during the study. A comprehensive Doppler ultrasound evaluation was conducted for all 60 patients in the study: once preoperatively, at two weeks postoperatively, and again at four weeks postoperatively. Across all 180 scans performed, no cases of thrombosis were detected in either the enoxaparin or long-term aspirin group, indicating a 0% incidence of VTE for both treatments. Figure 2 illustrates the comparison of VTE prevention rates between the two groups across various subcategories, including gender (women and men), surgery type (TKA and THA), obesity, diabetes, and overall thrombosis events. No statistically significant differences were observed between the groups in any category, as indicated by "ns" (non-significant). The primary outcome of this study comparing the 90-day incidence of postoperative thrombotic events and bleeding complications between the long-term aspirin and enoxaparin groups demonstrated the non-inferiority of aspirin.

## Discussion

Patients undergoing THA and TKA commonly receive chemoprophylaxis to prevent venous thromboembolism (VTE), a serious postoperative complication. While aspirin is a low-cost and widely available option, low-molecular-weight heparin (LMWH), specifically enoxaparin, is considered a standard prophylactic agent in many settings, albeit at a higher cost and with the inconvenience of daily injections [1,4-6]. Although the American Academy of Orthopedic Surgeons (AAOS) endorses the use of aspirin for thromboprophylaxis, the National Institute for Health and Care Excellence (NICE) in the United Kingdom does not universally support aspirin in this context, reflecting ongoing debate regarding its efficacy [9,16]. Additionally, Mexican healthcare guidelines have yet to issue specific recommendations for aspirin use in orthopedic patients.

This study hypothesized that an extended course of aspirin (100 mg/day orally for 28 days) following brief initial prophylaxis with enoxaparin (40 mg/day subcutaneously for 3 days) would be as effective and safe as a full 28-day regimen of enoxaparin in preventing VTE in patients undergoing TKA and THA, with potential advantages in cost-effectiveness. Our findings suggest that aspirin may indeed offer non-inferior efficacy to enoxaparin for VTE prevention, as no significant differences in thrombotic events were observed between groups. However, this conclusion must be interpreted cautiously due to the study's small sample size, which was a limitation recognized during the study design.

Our results align with previous research comparing aspirin to other anticoagulants for VTE prevention after arthroplasty. Chu et al., in a retrospective cohort study, demonstrated that aspirin could effectively prevent VTE in a subset of arthroplasty patients [14]. Large registry-based studies in the U.S., conducted by Moore et al. and Singh et al., reported that aspirin was associated with significantly lower VTE rates (0.4%) compared to other anticoagulants (1.2%-3.9%), with a high statistical significance (p < 0.0001) [17,18]. Additionally, Haykal et al.'s meta-analysis of randomized controlled trials revealed no significant difference in efficacy between aspirin and other anticoagulants (RR 0.87; 95% CI: 0.61-1.23; P = 0.43) [19].

EPCAT I and II trials further support extended aspirin prophylaxis after THA and TKA. EPCAT I demonstrated that a 28-day course of aspirin following 10 days of LMWH was a safe and effective alternative for VTE prevention [20]. EPCAT II, which included 3,424 patients, found aspirin non-inferior to rivaroxaban for VTE prevention when administered after an initial 5-day course of rivaroxaban [21]. Likewise, the most recent meta-analysis of randomized studies (86 studies) investigated the efficacy and safety profile of commonly used VTE prophylaxis agents following hip and knee arthroplasty by Cheok T et al. showed that aspirin <325 mg daily, enoxaparin, and dabigatran have an overall accepted efficacy and safety profile, they highlighted that aspirin <325 mg daily was most efficacious in reducing overall VTE with an acceptable risk profile. Although, they did not prevent fatal pulmonary embolism [22].

Additionally, another systematic review and meta-analysis focused on total knee arthroplasty (TKA) found no statistically significant differences in the prevention of deep vein thrombosis (DVT) (OR 0.93; 95% CI: 0.81-1.08; p = 0.35) or pulmonary embolism (PE) (OR 0.89; 95% CI: 0.56-1.41; p = 0.61) when comparing aspirin to other anticoagulants. This further substantiates the non-inferiority of aspirin in preventing thromboembolic events after TKA while also emphasizing its safety [23].

However, our findings differ from those of the CRYSTAL trial, which reported that enoxaparin was more effective than aspirin within 90 days postoperatively for preventing symptomatic VTE. Nonetheless, the CRYSTAL trial included a subgroup already on aspirin who continued aspirin if assigned to the enoxaparin arm, potentially impacting the outcomes [13].

One primary limitation of our study was the relatively small number of cases to detect differences in VTE rates between the two groups, the primary outcome of postoperative VTE complications was observed as zero in both groups. While the study was designed as a non-inferiority trial to compare the efficacy of aspirin and enoxaparin, it is important to acknowledge that a larger sample size would be required to observe such outcomes with greater statistical confidence. Additionally, the zero-event rate could be attributed to the use of effective perioperative protocols, including routine early mobilization and careful patient monitoring, which are known to reduce the incidence of VTE. However, this low event rate emphasizes the need for larger-scale studies to confirm the findings and provide a more robust evaluation of the interventions. Furthermore, this was a single-centre study. Another limitation was variability in adherence to the Doppler ultrasound follow-up, as some patients opted out (17 patients). Lastly, limited staffing resources restricted participant recruitment, resulting in a borderline sample size.

Despite these limitations, our study has several strengths. We randomized the participants into two groups to minimize selection bias. Unaware of the randomized group, one orthopedic surgeon was involved in all the cases to eliminate performance bias. Therefore, this study contributes to the growing body of evidence supporting aspirin as an effective, safe, and cost-efficient alternative to enoxaparin for VTE prophylaxis following joint arthroplasty. Only clinically significant events-symptomatic proximal deep vein thrombosis and pulmonary embolism- were considered outcomes, which were objectively confirmed through diagnostic testing. All participants were closely monitored for 90 days, ensuring the capture of any meaningful complications.

## Conclusions

This study found no significant difference in the prevention of VTE between extended low-dose aspirin and enoxaparin in patients undergoing total knee and hip arthroplasty. No symptomatic VTE events were observed in either group during the 90-day follow-up. These findings suggest that aspirin may be an effective and cost-efficient alternative to enoxaparin for thromboprophylaxis in this population.

However, the study's small sample size limits the ability to draw definitive conclusions. Future research should involve larger, double-blinded trials to more conclusively assess aspirin's efficacy relative to LMWH and other anticoagulants. Incorporating the insights from meta-analyses of clinical trials will be valuable in informing the design and scope of subsequent studies on this research. It will also help establish clearer guidelines and address variations in current recommendations among organizations like AAOS, NICE, and Mexican healthcare authorities.
